# Nutzenbewertung digitaler Gesundheitsanwendungen – Herausforderungen und Möglichkeiten

**DOI:** 10.1007/s00103-021-03413-x

**Published:** 2021-09-15

**Authors:** Lars G. Hemkens

**Affiliations:** 1 Basel Institute for Clinical Epidemiology and Biostatistics (ceb), Department of Clinical Research, University Hospital Basel, Spitalstrasse 12, 4031 Basel, Schweiz; 2 Meta-Research Innovation Center at Stanford (METRICS), Stanford University, Stanford, CA USA; 3 Meta-Research Innovation Center Berlin (METRIC-B), Berlin Institute of Health, Berlin, Deutschland

**Keywords:** Digital Health, Mobile Anwendungen, Nutzenbewertung, Pragmatische Studien, Studiendesign, Digital health, Mobile applications, Benefit assessment, Pragmatic trials, Research design

## Abstract

Digitale Gesundheitsanwendungen (DiGA) versprechen, die Gesundheit und medizinische Versorgung von Patienten zu verbessern. Dieser Beitrag gibt eine kurze Übersicht zur evidenzbasierten Nutzenbewertung und den Herausforderungen an die zugrunde liegende Evidenz als Voraussetzungen für eine optimale, patientenorientierte Entscheidungsfindung. Es werden klassische Konzepte des Studiendesigns, aktuelle Entwicklungen und innovative Ansätze beschrieben mit dem Ziel, zukünftige Entwicklungsfelder für innovative Studiendesigns und strategische Evaluationskonzepte für DiGA aufzuzeigen. Ein besonderer Schwerpunkt liegt auf der Anwendung pragmatischer Studiendesigns.

Evidenzbasierte Nutzenbewertung hat fundamentale Anforderungen und Maßstäbe unabhängig von der Art der evaluierten Behandlungen. Zuverlässige Evidenz ist unverzichtbar. Eine schnelle, effiziente, zuverlässige und praxisrelevante Evaluation von DiGA gelingt nicht durch eine Hinwendung zu nichtrandomisierten Studien, sondern vielmehr durch bessere, pragmatische randomisierte Studien. Sie sind machbar und verbinden die Charakteristika von DiGA, klassische methodische Konzepte und neue Ansätze der Studiendurchführung. Routinedaten, kontaktarme Studiendurchführung („virtual trials“, „remote trials“) und digitale Biomarker fördern nützliche randomisierte Real-World-Evidenz als solide Evidenzbasis von DiGA. Eine kontinuierliche, lernende Evaluation im Versorgungsalltag mit in die Routine eingebetteten randomisierten Studiendesigns ist der Schlüssel zur nachhaltigen und effizienten Nutzenbewertung von DiGA und kann entscheidend für eine strategische Verbesserung der Gesundheitsversorgung sein.

## Abstract (en)

Digital health applications promise to improve patient health and medical care. This analysis provides a brief overview of evidence-based benefit assessment and the challenges to the underlying evidence as prerequisites for optimal patient-oriented decision making. Classical concepts in study design, recent developments, and innovative approaches are described with the aim of highlighting future areas of development in innovative study designs and strategic evaluation concepts for digital health applications. A special focus is on pragmatic study designs.

Evidence-based benefit assessment has fundamental requirements and criteria regardless of the type of treatments evaluated. Reliable evidence is essential. Fast, efficient, reliable, and practice-relevant evaluation of digital health applications is not achieved by turning to nonrandomized trials, but rather by better pragmatic randomized trials. They are feasible and combine the characteristics of digital health applications, classical methodological concepts, and new approaches to study conduct. Routinely collected data, low-contact study conduct (remote trials, virtual trials), and digital biomarkers promote useful randomized real-world evidence as solid evidence base for digital health applications. Continuous learning evaluation with randomized designs embedded in routine care is key to sustainable and efficient benefit assessment of digital health applications and may be crucial for strategic improvement of healthcare.

## Einleitung

Digitale Technologien werden zunehmend zur Prävention, Diagnose oder Behandlung von Erkrankungen eingesetzt. Sie sind das Kernelement digitaler Gesundheitsanwendungen (DiGA), die zum Beispiel als Software auf Smartphones vielfältige Möglichkeiten versprechen, die Gesundheit und medizinische Versorgung von Patienten zu verbessern [1]. Seit 2019 können in Deutschland gesetzliche Krankenversicherungen die Kosten verordneter DiGA erstatten [1]. Wie bei jeder klinischen Entscheidung muss auch hier die Frage nach dem Nutzen und Schaden für Patienten gestellt werden.

Dieser Beitrag soll eine kurze Übersicht zur evidenzbasierten Nutzenbewertung und zu den Herausforderungen an die zugrunde liegende Evidenz als Voraussetzungen für eine optimale Entscheidungsfindung geben. Er beschreibt klassische Studiendesignkonzepte, aktuelle Entwicklungen und innovative Ansätze mit dem Ziel, zukünftige Entwicklungsfelder für innovative Studiendesigns und strategische Evaluationskonzepte für DiGA aufzuzeigen.

Der Beitrag fokussiert auf den gesundheitsbezogenen Nutzen oder Schaden, den Patienten bei der Behandlung von Krankheiten haben können, und daher nicht auf Primärprävention. Auf rechtliche, regulatorische, technologische und ökonomische Aspekte wird nicht eingegangen, ebenfalls nicht auf Rahmenbedingungen zur Zulassung oder Erstattung in bestimmten Gesundheitssystemen sowie Fragen des Datenschutzes oder der Informationssicherheit.

## Grundlagen der Nutzenbewertung

### Nutzen und Schaden

Wenn Patienten und Ärzte entscheiden müssen, welche Behandlungsoption die beste ist, haben sie als Entscheidungsgrundlage idealerweise optimale Informationen zu den erwartbaren Effekten auf für sie relevante Parameter (Endpunkte). Veränderungen von Lebensqualität, Morbidität und Mortalität sind unmittelbar patientenrelevant und zentral für Behandlungsentscheidungen [2, 3]. Der Nutzen einer medizinischen Handlung liegt in der Verbesserung dieser Endpunkte, ein Schaden in einer Verschlechterung.

Eine DiGA kann sich auf andere Parameter, z. B. die Strukturen und Prozesse der Gesundheitsversorgung auswirken. Hierfür wurde in Deutschland im Kontext von Erstattungsentscheidungen der Begriff „positiver Versorgungseffekt“ in Abgrenzung zu „Nutzen“ geprägt.

### Evidenzbasierte Entscheidungen

Bei der evidenzbasierten Entscheidungsfindung wird die Evidenz zum Nutzen und Schaden abgewogen unter Einbezug der jeweiligen Situation sowie der Wertvorstellungen und Präferenzen des Patienten [4]. Auf Methoden zur empirischen Bestimmung von Patientenpräferenzen wird an dieser Stelle nicht weiter eingegangen [5, 6].

Die prinzipiellen Anforderungen an evidenzbasierte Entscheidungsgrundlagen sind unabhängig von der Art der Behandlungen (z. B. Medikamente, chirurgische Therapie, DiGA), zwischen denen man sich entscheidet, und sollen eine zuverlässige Abschätzung von Nutzen und Schaden ermöglichen.

Theoretisch würden Entscheidungsgrundlagen Evidenz zur exakten Vorhersage der Folgen einer Handlung bzw. Behandlung und der jeweiligen Alternative bereitstellen und exakt den kausalen Zusammenhang zwischen Handlungsentscheidung und Ergebnis (Endpunkt) beschreiben [7]. In der Praxis verfügbare Evidenz ist hierin jedoch nur mehr oder weniger zuverlässig und wird mit ebenfalls mehr oder weniger Aufwand generiert. Dabei wird zunehmend erkannt, dass eine hohe Zuverlässigkeit nicht mit einem hohen Aufwand einhergehen muss [8].

## Klinisch-epidemiologische Grundlagen zu Evidenz und ihrer Zuverlässigkeit

### Randomisierung

Randomisiert kontrollierte Studien (engl. „randomized controlled trials“, RCTs) liefern die zuverlässigste Evidenz zum Nutzen und Schaden von Handlungsentscheidungen [9]. Denn sie erlauben es, kausale Effekte von Handlungen direkt abzuschätzen [7], und adressieren unmittelbar das kritische Problem von Verzerrungen des Zusammenhangs zwischen Exposition und Gesundheitsergebnis durch eine fremde dritte Variable (Confounding-Bias, s. unten). Die Randomisierung stellt 2 Vergleichsgruppen her, zwischen denen sämtliche Unterschiede (z. B. Risikofaktoren für einen schweren Krankheitsverlauf) zum Zeitpunkt der Entscheidung rein zufällig sind und daher recht einfach statistisch berücksichtigt werden können. In RCTs ist es nicht erforderlich zu wissen, welche Risikofaktoren relevant wären; entsprechende Daten müssen nicht erhoben werden, um eventuelle Dysbalancen zwischen Vergleichsgruppen zu suchen oder zu beschreiben [9]. Natürlich ist es oft hilfreich, Daten zu den Charakteristika der Studienpopulation zu haben, aber grundsätzlich ist es optional. In ihrer Reinform benötigen RCTs aus methodischer Sicht also nur die zufällige Zuteilung und die Messung von 2 Variablen – die Gruppenzugehörigkeit und einen Endpunkt.

### Keine Randomisierung

Nichtrandomisierte Studien haben den Vorteil der zufälligen Zuteilung nicht. Daher benötigen sie statistische Verfahren zur Adjustierung von Unterschieden zwischen den Vergleichsgruppen, um das Risiko für Bias durch Confounding bei fehlender prognostischer Balance zu verringern und so zumindest eine gewisse Vertrauenswürdigkeit zu gewährleisten [10]. Dazu muss man nicht nur sämtliche relevante Faktoren bzw. Confounder und ihre Interaktion kennen ([10–12]; was eine sehr starke und oft unrealistische Voraussetzung und auch eine subjektive Annahme ist), sondern man muss all diese Daten auch mit hohem Aufwand und hinreichend präzise sammeln (eine Aufgabe, die bei RCTs entfällt).

Für eine nichtrandomisierte Studie zum Effekt von DiGA auf Lebensqualität z. B. braucht es ein genaues Verständnis darüber, welche Faktoren mit Lebensqualität assoziiert sind und wie sie mit der Verordnung von DiGA zusammenhängen. Psychosoziale oder sozioökonomische Faktoren haben jedoch oft komplexe und unklare Zusammenhänge mit Lebensqualität, Morbidität oder Mortalität [13] und sind oft mit Therapieadhärenz und anderen Faktoren assoziiert [13]. Routinedaten für solche Faktoren sind selten vorhanden, was eine aktive (prospektive) Datensammlung mit erheblichem Mehraufwand (Datenmanagement, Qualitätsmanagement, Monitoring) erfordern würde [14]. Selbst in intensiv beforschten Bereichen herrscht zudem oft kein Konsens dazu, welche Faktoren bzw. Confounder auf welche Weise in Analysemodelle eingehen sollen [15].

Allerdings kann die Wahl des Modells die Ergebnisse massiv beeinflussen und durchaus eine geschätzte Risikoerhöhung in eine Risikosenkung verdrehen [16]. Diese im Vergleich zu RCTs ungleich höhere Vibration of Effects hat ein extremes Potenzial für bewusste oder unbewusste Einflussnahme und reduziert die Zuverlässigkeit weiter [12, 17, 18]. Hoch detaillierte Studienprotokolle und vorab festgelegte Analysepläne zum Schutz vor solchen Mechanismen wären essenziell, existieren jedoch nur selten – ganz im Gegensatz zur Situation bei RCTs, obwohl dort die Analysen sogar deutlich weniger komplex sind (da es diese Adjustierungen nicht braucht; [12]). Zahlreiche systematische metaepidemiologische Untersuchungen haben gezeigt, dass die Ergebnisse von RCTs und nichtrandomisierten Studien oft deutlich abweichen [12], auch wenn moderne statistische Verfahren verwendet werden (z. B. Propensity Scores, kausale Modelle; [17, 19]).

Insgesamt erlauben nichtrandomisierte Studien (oder gar Studien ohne Kontrollgruppe) keine verlässlichen Schlussfolgerungen zu Effekten von Therapieentscheidungen (von seltenen Situationen abgesehen; [9, 12]), während sie gleichzeitig oft einen hohen Zusatzaufwand mit sich bringen.

## Evaluation digitaler Innovationen mit randomisierten Studien außerhalb der Gesundheitsversorgung

Den Vorteil randomisierter Studien haben die erfolgreichsten Unternehmen der digitalen Wirtschaft (z. B. Microsoft, Google, Facebook, Amazon, Netflix) längst erkannt und nutzen sie systematisch zur kontinuierlichen Evaluation und Verbesserung ihrer Produkte, auch wenn ihnen große Datenmengen zur Verfügung stehen, die mitunter als Argument für nichtrandomisierte Studien angeführt werden [9, 20]. Tatsächlich finden die meisten RCTs heutzutage als sog. A/B-Tests in der digitalen Technologiebranche statt [20]. Anstatt Fortschritt zu behindern, beschleunigen sie Innovation und führen zu einer nachhaltigen Verbesserung wichtiger Kennzahlen (z. B. Umsatz oder Benutzerzufriedenheit; [20]). Jede noch so kleine Verbesserung der digitalen Produkte wird trotz der schnellen Innovationszyklen in kürzester Zeit einer systematischen, randomisierten Testung unterworfen [20]. Dies weist darauf hin, dass dies technisch und methodisch auch für DiGA möglich und vielversprechend wäre. Gleichzeitig zeigt es, dass Herausforderungen für schnelle Evaluationen, Anpassungen und Reaktionen nicht primär technisch-methodischer Natur sind, sondern sich z. B. aus regulatorischen Umständen ergeben.

Der nachhaltige Stellenwert für eine strukturelle Verbesserung des Gesamtsystems wird daran erkennbar, dass randomisierte Untersuchungen zahlreiche nutzlose Innovationen ausfiltern konnten, die zu keiner Verbesserung führten. Dies betraf 2 von 3 der vielversprechendsten Ideen, in manchen Bereichen lagen die Ausfälle im Bereich von 80–90 % [20].

## Herausforderungen: andere Studiendesigns oder andere randomisierte Studien?

Bei der Betrachtung der Evidenz zur Nutzenbewertung gilt es zu differenzieren, ob es andere Studiendesigns als RCTs braucht oder vielmehr andere RCTs [12]. Ein Großteil aller RCTs ist in der Tat ungeeignet, die Fragen zu beantworten, die für Entscheidungsträger, Kliniker und Patienten relevant sind, und kann daher in vielerlei Hinsicht als nutzlos angesehen werden [21]. Darauf wiesen Schwartz und Lellouch bereits 1967 hin [22]. Sie entwickelten das Konzept „explanatorischer“ und „pragmatischer“ RCTs, das zunehmend wieder in den Fokus gelangt. Für die Rahmenbedingungen zur Schaffung und Einordnung von Evidenz zur Nutzenbewertung von DiGA sind diese Konzepte essenziell.

### Explanatorische Studien

Zunehmend wird erkannt, dass die steigenden Herausforderungen bei der Durchführung von RCTs nicht am Studienprinzip liegen, sondern an der konzeptionellen Umsetzung neben gesundheitspolitischen und akademischen Rahmenbedingungen [9, 23]. Diese resultiert aus der Tradition der Arzneimittelforschung und hier vor allem der frühen Entwicklungsphase. Studien dieser Phase gleichen meist hoch kontrollierten Laborexperimenten und haben ein sogenanntes explanatorisches Design [22]. Sie wollen ein besseres Verständnis der pathophysiologischen Wirkmechanismen möglichst ohne Störeinflüsse durch z. B. suboptimale Therapieadhärenz liefern [24]. Placebos werden eingesetzt, um möglichst sauber den Effekt einer hoch standardisierten Verabreichung eines Wirkstoffes zu messen. Sie haben strenge Einschlusskriterien [25, 26] und viele Follow-up-Untersuchungen, um Variablen zu messen, die wichtig sein können, die Arzneimittel zu verbessern oder ihre Mechanismen zu verstehen, aber nicht unbedingt patientenrelevant sind (z. B. Biomarker oder Pharmakovigilanzdaten; [24]).

### Herausforderungen und Limitationen

Die meistgenannten Limitationen von RCTs ergeben sich direkt aus diesen Charakteristika explanatorischer Studien, ebenso die Herausforderungen bei der Durchführung von RCTs. Der hohe Aufwand erzeugt hohe Kosten, umso mehr, je größer und länger die Studie ist. Aus Kostengründen werden dann kleinere und kürzere Studien geplant, die es nicht erlauben, patientenrelevante Endpunkte und Langzeiteffekte zu messen oder relevante Subgruppeneffekte zu finden. Strikte Einschlusskriterien erschweren die Rekrutierung und können die Anwendbarkeit der Ergebnisse einschränken. Placebos erhöhen den Aufwand zudem mitunter beträchtlich (geschätzt 10 % des Gesamtstudienbudgets [27]) und führen zu einem Behandlungsvergleich, der unter Real-World-Bedingungen nicht existiert und reale Therapieentscheidungen nicht abbildet [24].

### Pragmatische Studien: Randomisierte Real-World-Evidenz

Für die Nutzenbewertung sind viele dieser Charakteristika nicht nur unnötig, sondern sogar kontraproduktiv. Studien, die Evidenz für Behandlungsentscheidungen liefern sollen, sollten diese strikten Eigenschaften explanatorischer Studien vermeiden [24]. Entscheidungsorientierte, sogenannte pragmatische RCTs haben die meisten traditionellen Limitationen von (explanatorischen) RCTs nicht. Dabei steht „pragmatisch“ keineswegs für niedrigere Ansprüche an Sorgfalt, Zuverlässigkeit oder für methodische Abkürzungen. Vielmehr steht die direkte Handlungsorientierung im Vordergrund. Pragmatische Studien liefern definitionsgemäß nützliche, patientenorientierte Evidenz, die direkt in Entscheidungen einfließen kann und möglichst wenig Annahmen zur Übertragbarkeit von der Studie auf die Praxis erfordert [24]. Sie finden entsprechend nicht unter artifiziellen Bedingungen mit selektierten Patienten und Placebokontrollen statt.

#### Machbarkeit

Die Machbarkeit hochwertiger pragmatischer RCTs zur kurzfristigen Bewertung neuer Interventionen hat das RECOVERY Platform Trial bei COVID-19 eindrucksvoll belegt. In 2 Tagen geplant, wurde nach 9 Tagen der erste und nach 2 Monaten der 10.000. Patient eingeschlossen [28, 29]. In kürzester Zeit wurden effektive Behandlungen gefunden und zahlreiche Therapien als nicht nutzbringend identifiziert (obwohl sie aufgrund mechanistischer Überlegungen plausibel und teilweise in großen nichtrandomisierten Studien vielversprechend waren; [28, 29]). Die Gründe für den Erfolg waren nicht nur die im Vereinigten Königreich solide etablierten Strukturen für klinische Studien und akademischen Rahmenbedingungen [23]. Entscheidend waren auch das sehr pragmatische Studiendesign, mit einer präzisen Reduktion der Prozesse auf das Wesentliche (z. B. mit lediglich 3 grundlegenden Einschlusskriterien), eine konsequente Einbettung in den Versorgungsalltag und die Nutzung von Routinedaten [28, 29].

#### Routinedaten

Routinedaten (z. B. aus Registern, Krankenhausdatenbanken oder Abrechnungsdaten) können die Durchführung von RCTs substanziell verbessern [8, 30, 31]. Statt aufwendig neue Dateninfrastrukturen aufzubauen, werden Daten benutzt, die sowieso erhoben werden. Das übrige Studiendesign eines RCT bleibt unverändert (d. h. eine randomisierte Zuordnung der Intervention), aber statt z. B. aktiv Erkundigungen einzuholen, ob ein Teilnehmer hospitalisiert war, wird z. B. der Versicherer angefragt. RCTs können auch vollständig in vorhandene Datenstrukturen eingebettet werden (z. B. Register oder Kohorten [31]) oder traditionelle Datenerhebung wird verknüpft mit Abfragen bestehender Datenquellen [32]. Mit Routinedaten können nicht nur Endpunkte gemessen, sondern auch Studienteilnehmer effizienter rekrutiert werden [8]. So werden RCTs zu einem Bruchteil der bisher üblichen Kosten möglich (in einer Größenordnung von 50–2000 USD pro Patient; [33]).

Darüber hinaus entfallen artifizielle Datenerhebungen und Interaktionen mit Teilnehmern nur für Studienzwecke und so ergibt sich eine größere Nähe zum normalen Versorgungsalltag. Routinedaten mit besonderer Bedeutung für DiGA sind solche, die z. B. von mobilen Geräten direkt gemessen werden (digitale Biomarker; s. unten). Natürlich muss eine hinreichende Qualität der Routinedaten gewährleistet sein, was jedoch zunehmend erwartet werden kann [14].

Insgesamt liefern pragmatische Studien naturgemäß eher die für eine Nutzenbewertung relevante Evidenz und sind ein Grundstein von Comparative Effectiveness Research bzw. Real World Evidence [32, 34, 35]. Sie vereinen hohe interne Validität von RCTs (hier stehen sie nichtpragmatischen RCTs in nichts nach) mit hoher externer Validität (Anwendbarkeit, Übertragbarkeit der Ergebnisse). Für die Evaluation von DiGA sind pragmatische RCTs daher zentral.

## Pragmatische Studien sind zentral für die Nutzenbewertung von DiGA

DiGA haben zahlreiche Charakteristika, die mitunter als Herausforderung für RCTs beschrieben wurden, aber bei näherer Betrachtung im Kontext von pragmatischen Studien keine oder nur eine geringe Rolle spielen. Viele Elemente explanatorischer Studien, die sich aus Parallelen zur frühen Arzneimittelentwicklung ergeben (z. B. Prüfungen der Verträglichkeit und Sicherheit, Dosisfindung), sind bei DiGA kaum relevant. Folglich treffen die Limitationen, die solche Elemente mit sich bringen für DiGA nicht zu. Weitere Elemente sind schon rein konzeptionell bei pragmatischen Studien nachrangig bis irrelevant.

### Ein- und Ausschlusskriterien

Die Studienpopulation entspricht in pragmatischen Studien der Zielpopulation, d. h. bei DiGA idealerweise den Patienten, denen sie verordnet werden würden. Ausschlüsse aus Sicherheitsgründen (z. B. wegen Begleiterkrankungen oder Arzneimittelwechselwirkungen) sind eigentlich nicht relevant. Eine artifizielle Homogenisierung der Studienpopulation (z. B. Ausschluss multimorbider Patienten) mit dem Ziel geringerer Effektvarianz und damit geringerer benötigter Fallzahl wäre hier explizit unerwünscht. Das erleichtert die Rekrutierung erheblich, senkt Kosten und Aufwand und erlaubt größere Studien in kürzerer Zeit.

### Adhärenz

Bei pragmatischen Studien steht explizit die Entscheidung für eine Therapieoption, die Intention der Behandlung, im Vordergrund und nicht die Durchführung bzw. Umsetzung [22, 24]. Wenn Teilnehmer trotz bester Intention eine Therapie nicht so befolgen wie angedacht oder gar niemals beginnen, ist dies Bestandteil der Behandlung als Ganzes [24]. Komplexe Wechselwirkungen mit dem Verhalten der Patienten beeinträchtigen die Zuverlässigkeit pragmatischer RCTs zum Einsatz von DiGA nicht, da sie der randomisierten Therapieentscheidung nachfolgen und so Bestandteile eines intendierten Therapiekonzeptes mit DiGA sind (entsprechend folgt die Analyse dem Intention-to-treat(ITT)-Prinzip; als sog. „Treatment Policy Estimand“ [17, 24, 36, 37]).

Bestimmt wird also der kausale Effekt der Intention der Behandlung und nicht ihre plangemäße Umsetzung [17, 24]. Dies ist plausibel, denn es ist z. B. wahrscheinlich nicht nützlich, eine DiGA zu verordnen, die unter Routinebedingungen fast nie benutzt wird, weil sie als zu umständlich wahrgenommen wird. Ebenso wäre die Verordnung eines abscheulich schmeckenden Hustentees, der nie getrunken werden würde, ohne Nutzen. Der Effekt einer perfekten Umsetzung ist für pragmatische Studien nicht relevant, da dies unter Real-World-Bedingungen unrealistisch und maximal artifiziell ist (dies wäre Gegenstand einer explanatorischen Studie).

Eingeschränkte Adhärenz, Behandlungs-Cross-over („Kontamination“) und Verhaltensinteraktionen erzeugen keinen Bias der ITT-Effekte in RCTs (s. oben; [24]). Auch entfällt für pragmatische RCTs die Notwendigkeit, detaillierte Daten zur Adhärenz zu erheben, was größere Studien realisierbarer macht und die Nähe zum Versorgungsalltag fördert. Gleichwohl lässt sich die Nutzung einer DiGA in vielen Fällen vergleichsweise einfach erfassen. So wurde in einer französischen pragmatischen RCT mit 2804 Teilnehmern zum Angebot einer Raucherentwöhnungs-App erkannt, dass sehr viele Teilnehmer die DiGA kaum oder gar nicht benutzt haben [38].

Eine Möglichkeit, den Nutzen bei Personen mit wahrscheinlich hoher Adhärenz zu testen, sind Run-in-Phasen zu Beginn einer Studie [39]. Patienten würde hier die DiGA testweise angeboten und die Studie würde diejenigen einschließen, die sie zumindest eine Zeit lang planmäßig verwenden. Da hier der Kontrollgruppe die DiGA vorenthalten würde (zumindest eine Zeit lang), könnte dies ein artifizielles Setting erzeugen und möglicherweise zu Unzufriedenheit und/oder Behandlungs-Cross-over führen (d. h., die Patienten beschaffen sich die DiGA selbst und wenden sie an, obwohl dies in der Studie nicht vorgesehen ist). Mit kausalen Modellen kann in RCTs versucht werden, die Effekte theoretisch optimaler Adhärenz zu messen (im Gegensatz zum ITT-Effekt; sog. Hypothetical Estimand; [36, 37, 40, 41]). Da hier jedoch die Randomisierung aufgegeben wird [40], bestehen wie bei nichtrandomisierten Studien ein sehr hohes Risiko für Bias, die Notwendigkeit, umfangreiche Daten zu sammeln, und weitere große Herausforderungen [41].

## Verblindung

Eine Verblindung von Arzt oder Patient ist in der üblichen Versorgung unrealistisch und erzeugt ein sehr artifizielles Behandlungssetting, was einem pragmatischen Studiendesign widerspricht. Die Zuverlässigkeit der Effektschätzer wird durch eine fehlende Verblindung nicht zwangsläufig beeinträchtigt, wenn analog den obigen Ausführungen zur Adhärenz argumentiert wird, dass die Kenntnis der Behandlung (mit allen sich daraus ergebenden Konsequenzen) nicht Bias erzeugt, sondern ein inhärenter Teil der Behandlungsentscheidung selbst ist [24]. Davon unbenommen ist die Notwendigkeit verblindeter Endpunkterhebung, da sonst ein Risiko für Bias bei der Ergebnismessung besteht. Eine Verblindung des Untersuchers bzw. der Studiendurchführung kann ggf. in gewissem Ausmaß vor bewusst verzerrenden, manipulativen Einwirkungen schützen (z. B. suboptimale Begleitbehandlungen der Kontrollgruppe). Routinedaten z. B. sind in der Regel formal verblindet, da die Erfassung von z. B. Hospitalisierungen durch Versicherer automatisch bzw. in Unkenntnis etwaiger DiGA-Verordnungen stattfindet. Die Verwendung subjektiver Endpunkte oder im Extremfall Patient-reported Outcomes (PROs), deren Messung sich durch die Kenntnis der Behandlung beeinflussen lässt, kann mit einem hohen Risiko für Bias einhergehen.

Somit ist die Verwendung von PRO-Endpunkten, wie z. B. Schmerzskalen oder Lebensqualität, bei der Evaluation von DiGA kritisch zu betrachten, wenn nicht tatsächlich adäquate Placebo-DiGA verwendet werden. DiGA zu verblinden ist nicht einfach (z. B. mittels Placebo-DiGA). Ob jedoch der im Versorgungsalltag nichtexistierende Vergleich mit Placebo-DiGA hilfreich ist, um Effekte auf z. B. Lebensqualität zu messen, muss diskutiert werden. Weniger artifiziell wäre ein Vergleich von 2 vom Patienten nicht unterscheidbaren DiGA-Versionen (die z. B. unterschiedliche Algorithmen anwenden).

## Besonderheiten bei der Evidenzgenerierung zu DiGA

Während die Prinzipien zur Nutzenbewertung und Evaluation von DiGA und anderen medizinischen Maßnahmen sich nicht unterscheiden und durch eine Hinwendung zu pragmatischen Studien auch traditionelle Hürden bei der Evidenzgenerierung ihre vermeintliche Relevanz verlieren, haben DiGA doch einige Besonderheiten (Tab. 1).

**Table 1:** 

Charakteristikum	Relevanz für Evidenzgenerierung	Relevanz für Nutzenbewertung	Anmerkungen
**DiGA-spezifisch**			
*Sicherheitsprofil* (geringes Risiko für schwere Nebenwirkung, keine strikte Indikationsstellung wegen Wechselwirkungen/Begleiterkrankungen)	Kaum Ausschlussgründe	Höhere Anwendbarkeit/Übertragbarkeit	Solide Festlegung des Sicherheitsprofils erfordert ebenfalls adäquate Evidenz
	Leichtere und schnellere Rekrutierung	Größere Studien (präzise Effektschätzer; ggf. Subgruppeninformationen)	
	Kontaktärmere Studie möglich^a^		
	Weniger Kosten und Aufwand		
	Größere Studie möglich		
	Leichtere Einbettung in Routineversorgung		
*Verfügbarkeit und Distribution* (Leichter Zugang, unmittelbarer Roll-out)	Einfachere Logistik	Größere Studien (präzise Effektschätzer; ggf. Subgruppeninformationen)	Skalierbarkeit oft leichter als bei Arzneimitteln (d. h. Kosten für DiGA oft weniger abhängig von Benutzerzahl)
	Kontaktärmere Studie möglich		
	Weniger Kosten und Aufwand		
	Größere Studie möglich		
	Leichtere Einbettung in Routineversorgung		
*Kurze Innovationszyklen* (Neue Versionen, sich verändernde Algorithmen)	DiGA kann sich während der Evaluation ändern: detaillierte Planung und Präspezifizierung nötig	Schnelle Bewertung notwendig	Reproduzierung von Studien mit obsoleten Versionen ggf. schwierig
	Plattform-Trials/adaptive Designs	Definition/Abgrenzung neuer Versionen notwendig	Follow-up ggf. eingeschränkt
*Digitale Biomarker* (Endpunkte durch digitale Geräte erhoben, neuartige Endpunkte)	Kontaktärmere Studie möglich^a^	Endpunktvalidierung notwendig	Endpunktmessung nur valide, wenn bei allen Studienteilnehmern gleich und unabhängig von der Gruppenzuteilung
	Erfordert digitale Kontrollintervention	Klärung der Patientenrelevanz	
*Verblindung* *(Placebo-DiGA)* (Arzt/Therapeut, Patient)	Wenn möglich zu vermeiden	Ggf. hohes Risiko für Bias bei subjektiven Endpunkten (z. B. PRO, LQ) und digitalen Biomarkern	Einfluss auf Bias sorgfältig diskutieren
	Unrealistisches Setting		
	Hoher Aufwand in der Umsetzung		
	Zusatzkosten durch Placebo		
	Nötig für digitale Biomarker und PRO		
*Verblindung (DiGA Version 1 vs. Version 2)* (Arzt/Therapeut, Patient)	Ggf. kein unrealistisches Setting	Bewertung subjektiver Endpunkte und digitaler Biomarker ggf. möglich	./
	Ggf. kein hoher Aufwand	Kontinuierliche Evaluation	
	Ggf. kaum Zusatzkosten		
	Verwendung digitaler Biomarker und PRO ggf. gut möglich		
	Zentral für lernende Evaluation		
**Pragmatische Studien generell**			
*Adhärenz* (inkl. Cross-over, Kontamination)	Nicht relevant (ITT-Analyse)	Kein Risiko für Bias in pragmatischen Studien	Daten zur Adhärenz könnten helfen, Wirkweisen der DiGA-Behandlung zu verstehen
	Keine aufwendige Datensammlung nötig		Run-in-Phasen sind ggf. hilfreich
	Keine spezielle Motivation (außerhalb der Routine) nötig und sinnvoll		
	Kontaktärmere Studie möglich		
	Weniger Kosten und Aufwand		
	Größere Studie möglich		
*Verblindung* (Endpunkterhebung)	Immer anzustreben	./	./

*ITT* Intention to Treat, *LQ* Lebensqualität, *PRO* Patient-reported Outcome

^a^Remote Trials/Virtual Trials

Vor allem die oftmals kurzen Innovationszyklen von DiGA werden gelegentlich als hinderlich für die Durchführbarkeit von RCTs angeführt. Die Technologien können sich so schnell verändern, dass neue Versionen bereits verfügbar sind, bevor die Bewertung des Vorgängers abgeschlossen ist. Ein Schlüssel zur Nutzenbewertung kann hier in einer kontinuierlichen, lernenden Evaluation sich kontinuierlich verändernder Varianten derselben DiGA liegen, die ständig (randomisiert) miteinander verglichen werden, ähnlich wie es heutzutage schon im nichtmedizinischen Bereich digitaler Technologie üblich ist [20].

Möglich wird eine solche kurzfristige Evaluation durch die in der Regel vorhandene schnelle Verfügbarkeit und Distribution neuer DiGA-Versionen, eine angemessene Studienplattform, eine hinreichende Dateninfrastruktur und ggf. weitere neue Ansätze zur Studiendurchführung.

### Verfügbarkeit und Distribution

Der logistische Aufwand bei der Distribution einer DiGA ist weniger komplex als bei Arzneimittelprüfungen, da DiGA online zum Anwender übertragen werden können und klassische Anforderungen an Transport, Lagerung, Bilanzierung des Verbrauchs etc. entfallen und Zeit, Kosten und Aufwand auch für Monitoring gespart werden. Gleichwohl ergeben sich andere Herausforderungen (Datensicherheit etc.). Entscheidend ist, dass langwierige logistische Planungen vermieden und sehr kurzfristig neuere Versionen einer DiGA zur Evaluation eingesetzt werden können.

### Lernende Evaluation im Versorgungsalltag

Eine zeitnahe und effiziente Evaluation verschiedener Versionen einer DiGA erfordert eine bereits vorhandene Studienplattform (Plattform-Trials bzw. Masterprotokolle [42, 43]) mit festgelegten grundsätzlichen Rahmenbedingungen der Evaluation (z. B. Einschlusskriterien, Endpunkte, statistische Analyse). Im detaillierten Studienprotokoll würde prädefiniert, ab wann kleinere Verbesserungen, Bugfixes und größere Updates tatsächlich einer neuen Version entsprechen [44]. Diese Versionen können dann in einer kontinuierlichen (adaptierten) Evaluation systematisch als hinzukommende Interventionen in zusätzlichen Studienarmen vergleichend untersucht werden, während andere Studienarme nach abgeschlossener Evaluation wegfallen. Eine kontinuierliche Weiterentwicklung im Rahmen der Anwendung kann erfolgen. Das ist eine im Vergleich zu herkömmlichen medizinischen Interventionen grundsätzliche und vielversprechende Neuerung. Sofern Unterschiede der Versionen für Nutzer nicht erkennbar sind (z. B. unterschiedliche Algorithmen), würde diese formale Verblindung eine Analyse subjektiver Endpunkte möglich machen.

#### Fixe oder verändernde DiGA.

Für die Nutzenbewertung muss klar festgelegt werden, was evaluiert wird – eine definierte DiGA mit einem fixen Algorithmus oder eine DiGA mit einem flexiblen, sich verändernden Algorithmus, der sich weiterentwickelt und sich möglicherweise auch dem Anwender individuell anpasst. Die Anpassungsfähigkeit eines Algorithmus könnte durchaus seine Stärke sein, weshalb eine Evaluation, die diese Charakteristika außer Acht lässt oder künstlich einschränkt, nicht hilfreich wäre. Therapiestrategien mit Anpassungen zu evaluieren ist in der klinischen Forschung keineswegs unüblich (z. B. kann sich die Anwendung eines chirurgischen Verfahrens auch über den Studienverlauf weiterentwickeln, wenn die Operateure lernen und besser mit einem neuen Verfahren vertraut werden [45]). Diese Erfahrung kann auf DiGA übertragen werden und besondere Methoden (inklusive Randomisierung) wurden vorgestellt [45, 46].

#### Remote Trials/Virtual Trials.

Kontaktarme Studien (sog. Remote Trials oder Virtual Trials) werden zunehmend erfolgreich durchgeführt [47–49]. Studien ohne Studienzentren sind bei DiGA eher möglich als bei anderen Maßnahmen, auch aufgrund der digitalen Distributionsmöglichkeiten. Breite Einschlusskriterien und nicht notwendige studienspezifische Untersuchungen zur Sicherstellung der Teilnehmersicherheit, die bei DiGA oft eher möglich sind als bei experimentellen Arzneimitteln, fördern zudem eine effiziente und zügige Rekrutierung auch ohne Studienzentren. Die Interaktion mit Teilnehmern erfolgt weitgehend virtuell, ohne regelmäßige Visiten der Studienzentren [49]. So gelingt eine weitreichende, ortsungebundene Rekrutierung. Die Datenerfassung kann direkt online mit sicheren Systemen durch die Teilnehmer erfolgen, aufwendiges Monitoring würde entfallen. Der oben genannte RCT zur Raucherentwöhnung z. B. rekrutierte Teilnehmer landesweit online über die Website der französischen gesetzlichen Krankenkasse. Endpunkte wurden über Onlinefragebögen erfasst. Jedoch bestehen durchaus Herausforderungen, die sorgfältig untersucht werden müssen (z. B. wenn besondere regulatorische Anforderungen oder manche Untersuchungen direkten Kontakt mit Studienpersonal erfordern; [47–49]). Insgesamt kann dieser Ansatz wegbereitend sein für ein lernendes Evaluationsmodell von DiGA.

#### Digitale Biomarker.

Smartphones (und andere mobile Geräte) erlauben eine schnelle und kontinuierliche Messung von Informationen, die Gesundheit reflektieren und Endpunkte von Studien zur Nutzenbewertung sein können. Diese Informationen können weit komplexer und granularer sein als traditionelle Endpunkte, z. B. durch häufigere und zeitlich präzisere Messungen [50]. Sie sind als digitale Biomarker ein vielversprechendes Zukunftsfeld, z. B. um Krampfanfälle oder kardiale Arrhythmien zu erkennen [51], aber könnten möglicherweise auch helfen, Patienten zu identifizieren, die von einer Therapie besonders profitieren. Eine valide Verwendung setzt allerdings eine für alle Studienteilnehmer gleiche Messung (und gleiche Wahrscheinlichkeit für fehlende Werte) voraus. Sie darf in keinem Zusammenhang mit der DiGA stehen. Große Herausforderungen bestehen bei Kontrollen ohne DiGA, während digitale Biomarker für einen direkten Vergleich zweier DiGA-Versionen im Rahmen einer lernenden Evaluation sehr großes Potenzial haben.

## Fazit

Evidenzbasierte Nutzenbewertung hat fundamentale Anforderungen und Maßstäbe unabhängig von der Art der evaluierten Behandlungen. Zuverlässige Evidenz ist unverzichtbar. Eine schnelle, effiziente, zuverlässige und praxisrelevante Evaluation von DiGA gelingt nicht durch eine Hinwendung zu nichtrandomisierten Studien, sondern vielmehr durch bessere, pragmatische RCTs. Sie sind machbar und verbinden die Charakteristika von DiGA, klassische methodische Konzepte und neue Ansätze in der Studiendurchführung. Routinedaten, kontaktarme Studiendurchführung und digitale Biomarker fördern nützliche randomisierte Real-World-Evidenz als solide Evidenzbasis von DiGA. Besondere Herausforderungen ergeben sich bei der verlässlichen Messung der Endpunkte in oft unverblindeten Studien und der Datenqualität. Insgesamt ist eine kontinuierliche, lernende Evaluation im Versorgungsalltag mit in die Routine eingebetteten, randomisierten Studiendesigns der Schlüssel zu einer nachhaltigen und effizienten Nutzenbewertung von DiGA und kann entscheidend für eine strategische Verbesserung der Gesundheitsversorgung sein.
